# Human placental exosomes induce maternal systemic immune tolerance by reprogramming circulating monocytes

**DOI:** 10.1186/s12951-022-01283-2

**Published:** 2022-02-18

**Authors:** Kunfeng Bai, Cheuk-Lun Lee, Xiaofeng Liu, Jianlin Li, Dandan Cao, Li Zhang, Duanlin Hu, Hong Li, Yanqing Hou, Yue Xu, Anita S. Y. Kan, Ka-Wang Cheung, Ernest H. Y. Ng, William S. B. Yeung, Philip C. N. Chiu

**Affiliations:** 1 Department of Obstetrics and Gynaecology, LKS Faculty of Medicine, The University of Hong Kong, Hong Kong S.A.R., China; 2 The University of Hong Kong Shenzhen Key Laboratory of Fertility Regulation, Shenzhen, China; 3 Department of Obstetrics and Gynaecology, The University of Hong Kong-Shenzhen Hospital, Shenzhen, China

**Keywords:** Maternal immune tolerance, Placental exosome, Monocyte, T cell, PD-L1, PTEN

## Abstract

**Background:**

The maternal immune system needs to tolerate the semi-allogeneic fetus in pregnancy. The adaptation occurs locally at the maternal–fetal interface as well as systemically through the maternal circulation. Failure to tolerate the paternal antigens may result in pregnancy complications, such as pregnancy loss and pre-eclampsia. However, the mechanism that regulates maternal immune tolerance, especially at the systemic level, is still an enigma. Here we report that the first-trimester placenta-derived exosomes (pEXOs) contribute to maternal immune tolerance by reprogramming the circulating monocytes.

**Results:**

pEXOs predominantly target monocytes and pEXO-educated monocytes exhibit an immunosuppressive phenotype as demonstrated by reduced expression of marker genes for monocyte activation, T-cell activation and antigen-process/presentation at the transcriptomic level. They also have a greater propensity towards M2 polarization when compared to the monocytes without pEXO treatment. The inclusion of pEXOs in a monocyte-T-cell coculture model significantly reduces proliferation of the T helper cells and cytotoxic T cells and elevates the expansion of regulatory T cells. By integrating the microRNAome of pEXO and the transcriptomes of pEXO-educated monocytes as well as various immune cell functional assays, we demonstrate that the pEXO-derived microRNA miR-29a-3p promotes the expression of programmed cell death ligand-1, a well-known surface receptor that suppresses the adaptive immune system, by down-regulation of phosphatase and tensin homolog in monocytes.

**Conclusions:**

This is the first report to show how human pEXO directly regulates monocyte functions and its molecular mechanism during early pregnancy. The results uncover the importance of pEXO in regulating the maternal systemic immune response during early pregnancy by reprogramming circulating monocytes. The study provides the basis for understanding the regulation of maternal immune tolerance to the fetal allograft.

**Graphical Abstract:**

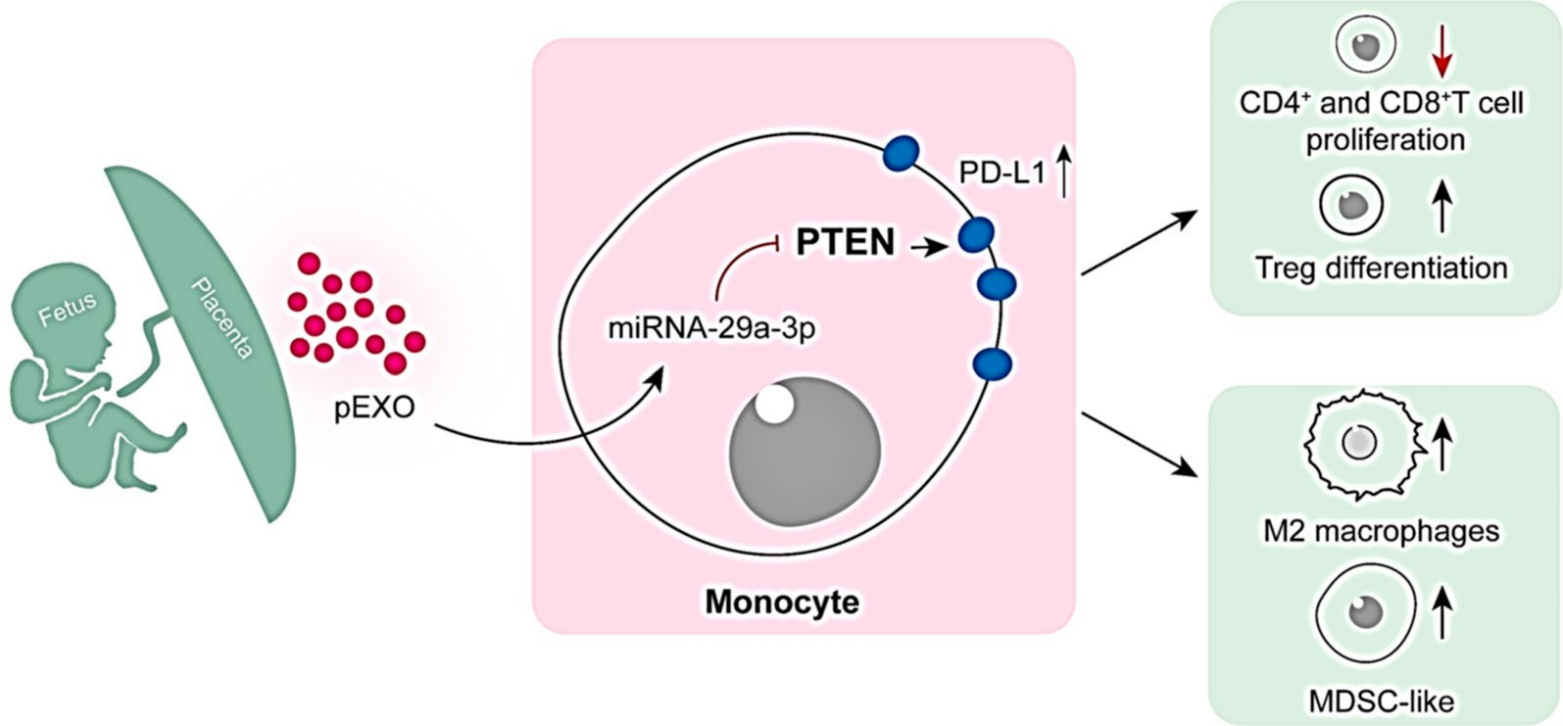

**Supplementary Information:**

The online version contains supplementary material available at 10.1186/s12951-022-01283-2.

## Background

Maternal tolerance towards the semi-allograft fetus is fundamental to a successful pregnancy. The mother’s immune cells encounter fetal antigens at two distinct sites: maternal–fetal interface starting 5–6 days after fertilization [1] and fetal villi bathing in maternal blood in the intervillous space after 9 weeks of gestational age when the utero-placenta circulation is established [2]. Since the maternal immune cells are in direct contact with the fetal semi-allogeneic trophoblast cells, adaptations must be established in the maternal immune system to avoid detrimental immune responses against the allogeneic fetus. In decidua, the immune cells undergo a phenotypic adaption and redistribution resulting in the accumulation of pregnancy-supporting natural killer (NK) cells and macrophages and reduction in the proportion of T cells, B cells and dendritic cells (DCs) [1]. Adaptations in the maternal systemic immune response are also observed, such as a decreased T helper cell type (Th)1/Th2/Th17 ratio [3] and increased numbers of regulatory T cells (Tregs) in the maternal circulation during the first and second trimester of pregnancy [4].

Accumulating data suggest that altered functional activity of monocyte–macrophage system is involved in dysfunctional maternal tolerance in pregnancy-related complications, such as implantation failure, pregnancy loss, preeclampsia and fetal growth restriction (FGR) [5]. Monocytes, the major phagocytic cell population in the systemic circulation, are critical mediators of innate and adaptive immune responses. They represent ~ 10% of the leukocytes in human circulation [5]. In normal pregnancy, there are functional changes in the circulating monocytes of mothers, including increased production of oxygen free radicals and changes in cytokine production [6]. From the start of pregnancy, monocytes are recruited to the maternal–fetal interface where they differentiate to decidual macrophages (dMs), which regulate maternal immune tolerance and promote placentation through interaction with other immune cells and fetal trophoblasts [7].

The exact mechanisms by which pregnancy-induced monocyte functional changes are unknown. It has been suggested that the factors released by trophoblasts such as cytokines [8], fetal DNAs [9], or hormones contribute to the changes in monocytes [6]. Extracellular vesicles are a newly found mode of intercellular communications. Exosomes are involved in a wide range of autocrine and paracrine phenomena [10]. They are nanoparticles (30–200 nm in diameter) formed by the inward budding of endosomal membrane. The bilayer structure of exosomal membrane protects the cargoes from degradation in extracellular environment. During pregnancy, placenta-derived exosomes (pEXOs) are shed from the syncytiotrophoblast into the circulation and their concentration in maternal blood increased as pregnancy proceeds [11, 12]. However, their biological role in pregnancy is poorly understood. Some evidence suggests that they are important in maintaining maternal tolerance, as pEXO promotes T cell apoptosis and inhibit cytotoxicity of NK cells [11, 12].

Successful pregnancy is associated with the functional regulation of monocytes [9, 11]. We hypothesized that the pEXO facilitates the establishment of maternal immune tolerance via regulating monocyte phenotype in early pregnancy. In this study, pEXOs were isolated from the first trimester placenta explants, and monocytes were demonstrated to be the main immune cell type in blood that could uptake pEXO. By integrating the first miRNA expression profiles of pEXO and the gene-expression data of pEXO-educated monocytes, together with the immune cell functional assays, we demonstrated for the first time that pEXO is a key regulator of maternal systemic immune response in early pregnancy by programming circulating monocytes via miRNA-29a-3p/phosphatase and tensin homolog (PTEN)/programmed cell death ligand-1 (PD-L1) axis. The outcome of the study enhances our understanding of the establishment of the maternal immune tolerance to the fetal allograft. In the long term, the study proposed that pEXOs might have the potential to serve as a biomarker for the diagnosis and treatment of immune-associated pregnancy complications.

## Results

### Purification of placenta-derived exosomes

**Fig. 1:**
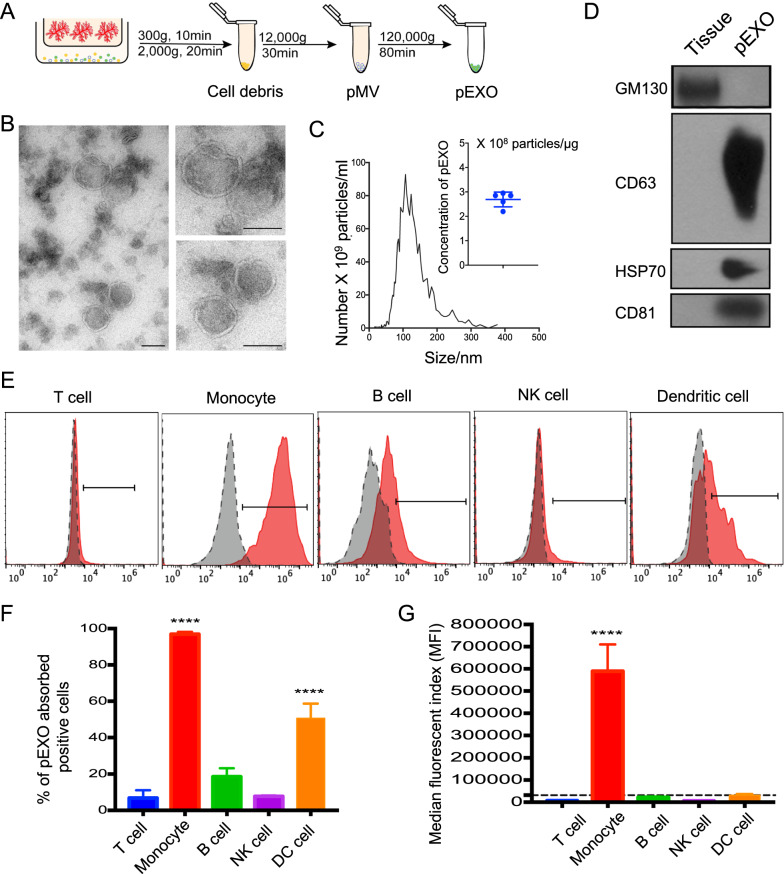
Characterization of pEXOs and interaction by different immune cell populations. **A** Schematic illustration of the sequential centrifugation to isolate exosomes from conditioned medium of human placenta explant. **B** Transmission electron microscopy images of pEXO, Scale bar = 100 nm. **C** NTA of the isolated pEXO. Particle concentration of pEXO is approximately 2.75 × 10^8^ particles/μg exosome protein. The mean size is 113 nm. **D** Western blot analysis demonstrates the presence of exosome markers CD63, HSP70, CD81 and the absence of GM130 (Golgi marker). **E** Flow cytometric analysis demonstrating the interaction of fluorescently labelled pEXO with different immune cell populations after 24 h. pEXO were mainly interacted with CD14^+^ monocytes. B cells and dendritic cells have a mild interaction with pEXO, while T cells and NK cells have barely no interaction. **F** Percentage of Carboxy-fluorescein succinimidyl ester (CFSE)-labelled pEXO positive cells and **G** Median fluorescent index (MFI) in different immune cell populations. Data are expressed as mean ± SD (n = 4), *p < 0.05, **p < 0.01, ***p < 0.001, ****p < 0.0001 compared to the control group

To study the biological roles of exosome, it is essential to establish an approach to obtain high-quality and physiological exosome from a consistent and reliable source [11]. Human first trimester placental explants were cultured for 40 h, and pEXOs were isolated from the spent media of placenta explants by differential centrifugation (Fig. 1A). Consistent with the International Society of Extracellular Vesicles guideline and other studies [13], the isolated pEXOs displayed a typical round morphology under transmission electron microscopy (Fig. 1B) and an averaged diameter of 113 nm by nanoparticle tracking analysis (NTA). There were about 2.69 × 10^8^ pEXOs per microgram total protein (Fig. 1C). They were also positive for exosomal markers CD63, HSP70 and CD81, and negative for Golgi marker, GM130 (Fig. 1D).

### Monocytes are the main immune cell type that interact with pEXO

To investigate the interactions between pEXOs and circulating immune cells, fluorescent-labelled pEXOs were incubated with PBMC. pEXO-interacted T cells, B cells, NK cells, DCs, and monocytes were characterized and gated with CD3, CD19, CD56, CD11c, and CD14 (Additional file 1: Fig. S1). Flow cytometry analysis demonstrated that the monocytes exhibited the highest median fluorescent index and were the major cell type interacting with the pEXOs (Fig. 1E–G). In contrast, T cells and NK cells had minimal interaction with pEXOs. DCs were the second major immune population in the interaction. However, the median fluorescent index of DCs was less than 10% of that of the monocytes (Fig. 1F, G). These results indicated that the pEXO mainly targets the monocytes in the maternal blood.

### pEXO-educated monocytes exhibited an immunosuppressive phenotype at the transcriptomic level

**Fig. 2:**
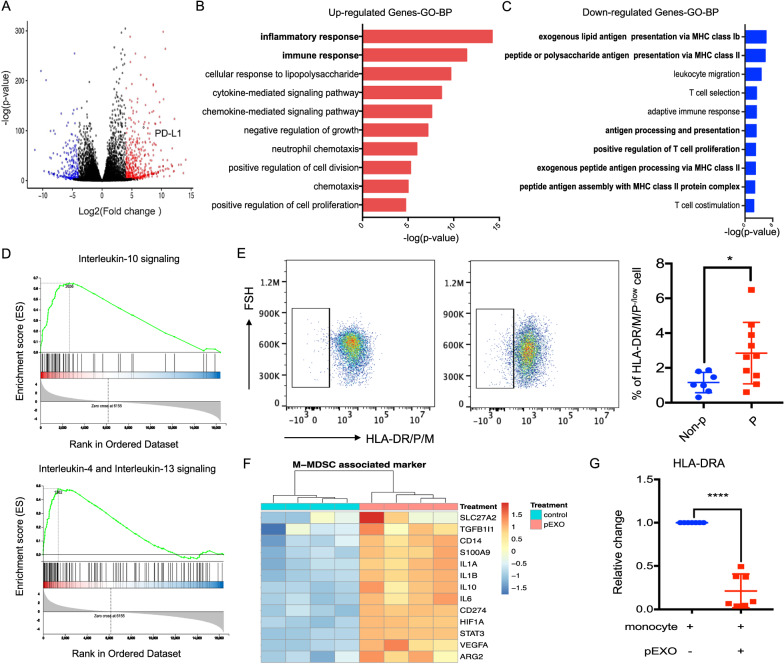
Transcriptional regulation of pEXO-educated monocytes. Monocytes were treated with 20 μg/ml pEXO for 24 h and subject to mRNA sequencing. **A** Volcano plot of differential expressed genes (DEGs, fold change > 16, Qvalue < 0.001) in pEXO-educated monocytes. Dots represent genes, colored as: not significant (NS, dark grey); UP (red) with log2FC ≥ 4.0 and adjusted p ≤ 0.001; DOWN (blue) with log2FC ≤ − 4.0 and adjusted p ≤ 0.001. p values are based on a two-tailed Wald test and adjusted via the Benjamini–Hochberg procedure. **B** GO analysis of up-regulated genes. **C** GO analysis of down-regulated genes. **D** Hallmark Gene Set enrichment analysis of pEXO-educated monocytes showing induction of gene sets regulating IL-10, IL-4 and IL-13 signaling in pEXO-educated monocytes. **E** Flow cytometry analysis of CD14^+^HLA-DR/DM/DP^−/low^ monocytes from pregnant women and non-pregnant control. N = 11, each dot represents one sample. Non-p: non-pregnant; P: pregnant. **F** The expression of MDSC marker, SLC27A2, TGFB1, CD14, S100A9, IL-1α, IL-1β, IL-10, IL-6, CD274, HIF-1A, STAT3, VEGF-A and ARG2 in pEXO-educated monocytes. **G** Reduction of HLA-DR at mRNA level in pEXO-educated monocyte (n = 8). Data are expressed as mean ± SD (n = 4), *p < 0.05, **p < 0.01, ***p < 0.001, ****p < 0.0001 compared to the control group

The selective interaction of pEXOs might modify the transcriptome and thus the functions of the target cells. Therefore, we examined the transcriptomic changes of pEXO-educated monocytes after 24 h of treatment. A total of 4864 differentially expressed genes (DEGs) were identified with 2324 upregulated and 2540 downregulated genes (fold change > 2, Qvalue < 0.05) in the pEXO-educated monocytes compared to the untreated control. DEGs with fold change > 16, Qvalue < 0.001 were retrieved for further analysis (Fig. 2A and Additional file 2). Gene ontology (GO) enrichment analyses revealed that most of the up-regulated genes were associated with inflammatory response, cytokine signaling pathway, and regulation of cell proliferation (Fig. 2B). The down-regulated genes were primarily associated with antigen processing and presentation, T cell proliferation, and differentiation (Fig. 2C). Notably, gene set enrichment analysis (GSEA) showed marked enrichment of the REACTOME interleukin (IL)-10 signaling pathway (Rich ratio = 0.74, FDR q = 0) and IL-4 and IL-13 signaling pathway (Rich ratio = 0.49, FDR q = 0) (Fig. 2D).

Further analysis revealed that monocyte activation markers CD36, CD74, CD200R and other immune response-related genes were significantly down-regulated in the pEXO-educated monocytes (Additional file 1: Fig. S2A). We also found that the antigen-processing and presentation associated genes, including CD1A, CD1B, CD1C, CD1D, CD1E and major histocompatibility complex (MHC) class II molecules such as human leukocyte antigen (HLA)-DR, HLA-DM, HLA-DP and HLA-DO were significantly suppressed in the pEXO-educated monocytes (Additional file 1: Fig. S2B, C). Consistently, the pEXO-educated monocytes displayed a decreased expression of genes that were positively correlated with T cell proliferation and activation, and an enhanced expression of genes that were negatively correlated with T cell proliferation and activation (Additional file 1: Fig. S2D). Together, these data indicated that the circulating monocytes display a tolerogenic and immuno-suppressive phenotype after pEXO treatment.

### pEXO induces CD14^+^HLA-DR^−/low^ monocytic myeloid-derived suppressor cells (M-MDSCs) expansion

CD14^+^HLA-DR^−/low^ M-MDSCs are monocytes with potent immunosuppressive activity which have attracted a lot of attention in the field of immunology in recent years [14]. They are capable of inhibiting T-cell responses and are highly increased in the early stages of pregnancy [14, 15]. Consistently, our data showed that the circulating CD14^+^HLA-DR^−/low^ M-MDSC population was significantly higher in pregnant women when compared to the non-pregnant control (Fig. 2E). Interestingly, 13 out of 16 genes that can distinguish M-MDSC from monocytes were up-regulated by pEXOs at mRNA level (Fig. 2F). The inhibitory effect of pEXOs on HLA-DRA expression in monocytes was confirmed by qPCR (Fig. 2G). The results indicated that pEXO is involved in regulating M-MDSCs expansion.

### pEXO modulates the M2 (alternatively activated) macrophage differentiation

**Fig. 3:**
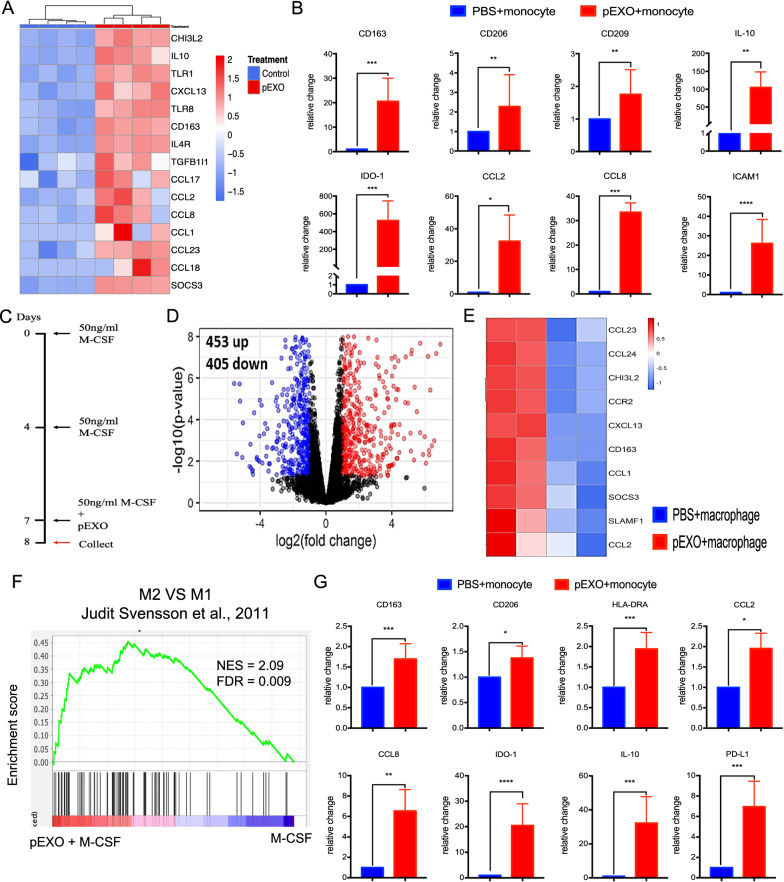
pEXO treatment reprograms CD14^+^ monocytes to an M2 macrophage phenotype. **A** Heatmap showing M2 markers were elevated in pEXO-educated monocytes. **B** Upregulation of M2-macrophages markers, CD163, CD206, CD209, IL-10, IDO-1, CCL-2 and CCL-8; cell adhesion molecule ICAM-1 and down-regulation of antigen-presenting molecules HLA-DRA were validated by RT-qPCR. (N = 8). **C** pEXO promoted macrophage polarization toward an M2 phenotype in human monocyte-derived macrophages. Schematic illustration of the strategy of human monocyte-derived macrophage and treatment. To induce macrophages polarization, monocytes were treated with 50 ng/ml M-CSF for 7 days and the medium was refreshed on DAY 4. Cells were harvested after 24 h of treatment with 20 ug/ml pEXO on day 7. **D** Volcano plot of differential expressed genes of the pEXO-polarized macrophages. **E** Heatmap of 10 M2 markers in pEXO-polarized macrophages compared to control group. **F** Gene set enrichment analysis (GSEA) with published M2 macrophage signature gene set comparing pEXO-polarized and control macrophages. **G** M2 macrophage markers: CD163, CD206, CD209, IL-10, CCL-2, CCL-8, IDO-1, and HLA-DRA of pEXO-polarized and control macrophages were determined by RT-qPCR. Data are expressed as mean ± SD (n = 8–12). *p < 0.05, **p < 0.01, ***p < 0.001, ****p < 0.0001 compared to the control group

pEXO-educated monocytes displayed M-MDSC characteristics (Fig. 2F) and enhanced expression of M2 macrophage markers, such as CD163, IL-10, CD206, CD209, IL-10, IDO-1 as demonstrated by mRNA-seq (Fig. 3A) and qPCR (Fig. 3B). These results suggested monocytes showed an M2 macrophage phenotype after pEXO treatment. To assess whether pEXOs affect macrophage differentiation, CD14^+^ monocytes were differentiated into macrophages with macrophage colony-stimulating factor (M-CSF) stimulation in the presence (pEXO-polarized macrophage) or absence of pEXOs (control macrophage) (Fig. 3C). mRNA-seq data demonstrated that M2 macrophage markers (CCL1, CCL2, CCL23, CCL24, CCR2, CHI3L2, CXCL13, CD163, SOCS3, SLAMF1) were significantly increased in the pEXO-polarized macrophages when compared to the control macrophages (Fig. 3D, E, Additional file 3). Consistently, GSEA revealed that the pEXO-polarized macrophages resembled the M2a macrophages described in the published data [16] (Fig. 3F). The bioinformatics analysis was confirmed by RT-PCR analysis of the M2 macrophage marker genes (CD163, CD206, CD209, IL-10, IDO-1, CCL2, CCL8) in the treatment group (Fig. 3G). Taken together, our data indicated that the pEXO promoted M2 macrophage differentiation of blood monocytes.

### pEXO-educated monocytes influence T cell proliferation and Treg expansion

**Fig. 4:**
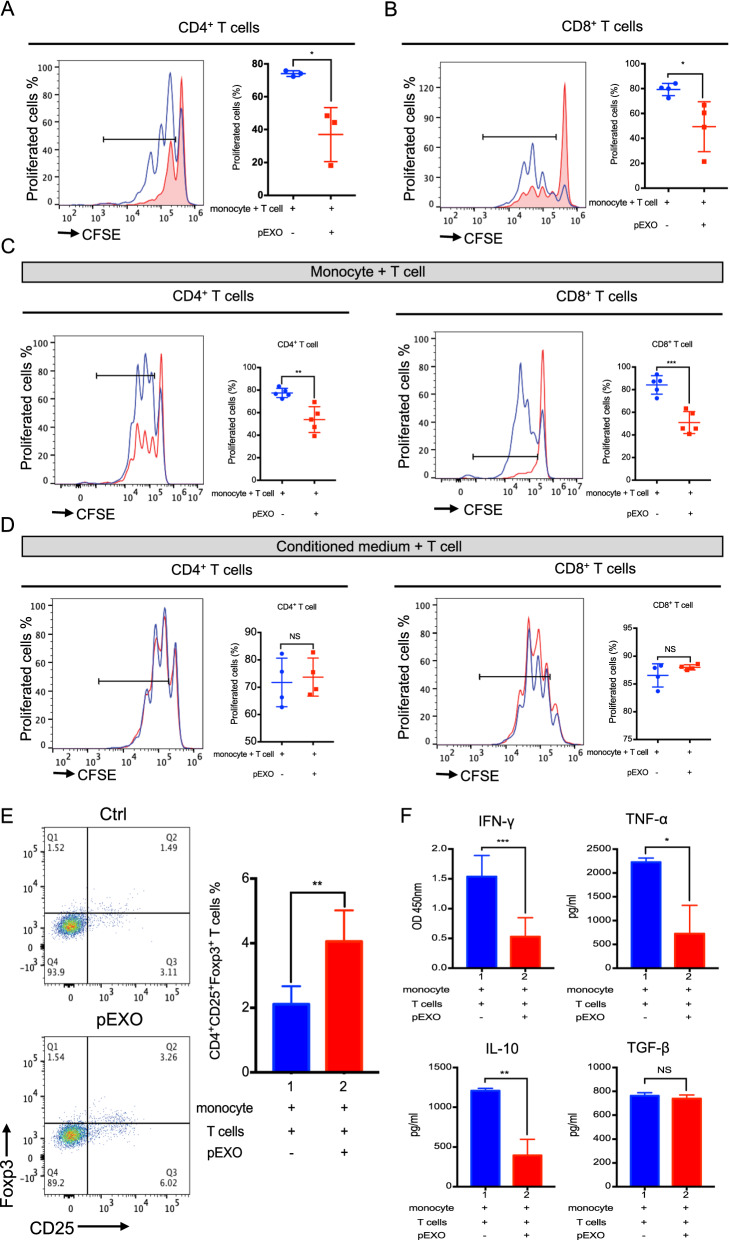
pEXO-educated monocytes facilitated immune tolerance by mediating T cell functions. **A**, **B** Representative histogram of CFSE-labelled CD4^+^ T cell and CD8^+^ T cell proliferation; CFSE-fluorescently labelled autologous T cells co-culture with CD14^+^ monocyte in the presence/absence of 20 μg pEXO treatment in a ratio of 1:1. **C** pEXO-educated monocyte inhibited autologous T cell proliferation in a direct co-culture model. **D** Conditioned medium collected from pEXO-educated monocytes had no effect on autologous T cells proliferation. **E** Flow cytometric analysis showing the increased population of Treg cells (CD4^+^CD25^+^Foxp3^+^) in co-culture of autologous T cells and pEXO-educated monocytes. **F** The cytokine profile of supernatant of monocyte-T cells co-culture was analyzed by ELISA (IFN-γ, TNF-α, IL-10 and TGF-β). Data are expressed as mean ± SD (n = 4). *p < 0.05, **p < 0.01, ***p < 0.001, ****p < 0.0001 compared to the control group

T cells, the major cell population involved in cell-mediated immune response, play a crucial role in maternal–fetal immune tolerance. An excessive inflammatory microenvironment by the up-regulation of effector T cells, such as CD3^+^CD4^+^ T helper type 1 (Th1) and CD3^+^CD8^+^ cytotoxic T cell, is associated with reproductive failures [3]. Monocytes alone did not influence autologous T cell proliferation (Additional file 1: Fig. S3A). In contrast, the inclusion of pEXOs significantly reduced the proliferation of CD3^+^CD4^+^ and CD3^+^CD8^+^ T cells in comparison to the untreated control (Fig. 4A, B). pEXO treatment had no effect on viability and/or apoptosis of human monocytes (Additional file 1: Fig. S3A) and CD3^+^CD4^+^/CD3^+^CD8^+^ T cells (Additional file 1: Fig. S3B). Direct co-culture with, but not the spent medium of pEXO-educated monocytes, decreased the CD3^+^CD4^+^ and CD3^+^CD8^+^ T cell proliferation in the presence of pEXO (Fig. 4C, D). Together, the data indicated that the pEXO-induced suppression of T cell proliferation required direct T cell-monocyte contact.

The numbers of systemic CD4^+^CD25^+^FoxP3^+^ Tregs increase during the first and second trimester of pregnancy [17]. To determine the role of pEXO in Tregs induction, T cells were co-cultured with monocytes for 3 days in the presence of pEXO. Flow cytometry analysis showed that the frequency of CD4^+^CD25^+^FoxP3^+^ Tregs was doubled when the cells were co-cultured with pEXO-educated monocytes (Fig. 4E). Consistently, cytokine profiling showed constrained secretion of cytotoxic cytokines, IFN-γ and TNF-α, in the spent medium of pEXO-educated monocytes-T cell co-culture (Fig. 4F).

### pEXO triggers PD-L1 expression via PTEN signaling pathway in human monocytes

**Fig. 5:**
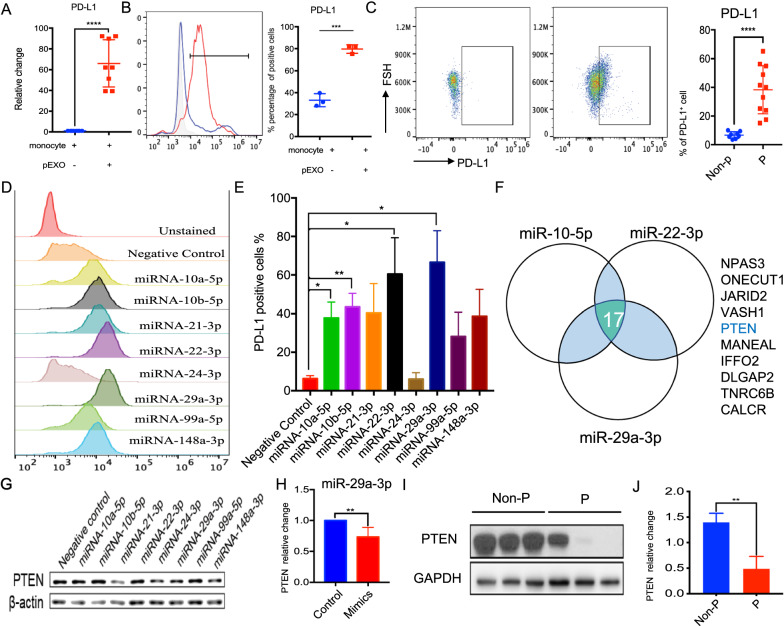
miRNA-29a-3p enhanced PD-L1 expression by targeting PTEN expression. **A** Enhanced expression of PD-L1 at mRNA level in pEXO-educated monocyte (n = 8). **B** Flow cytometry analysis of the expression of PD-L1 on pEXO-educated monocytes. N = 3, each dot represents one sample. **C** Increased frequencies of CD14^+^PD-L1^+^ monocytes from pregnant women and non-pregnant control. (Right). N = 11, each dot represents one sample. Non-p: non-pregnant; P: pregnant. **D** Representative histogram of PD-L1 expression in monocyte after miRNAs transfection. **E** Quantitative analysis of PD-L1 level in monocyte after miRNAs transfection. **F** Overlapped target genes between miRNA-10-5p, miRNA-22-3p and miRNA-29-3p by TargetScan 7.2 predication. **G** Western Blot analysis of PTEN expression after miRNAs mimics transfection. **H** Quantitative analysis of PTEN expression 24 h after miRNA-29a-3p mimics transfection. **I** The level of PTEN in monocytes of pregnant women and non-pregnant women (P & Non-P). **J** Quantitative analysis of PTEN expression of the circulating monocytes of pregnant women and non-pregnant women. (N = 3) *p < 0.05, **p < 0.01, ***p < 0.001, ****p < 0.0001 compared to the control group

Programmed cell death ligand-1 (PD-L1) is one of the modulators in peripheral tolerance and Tregs differentiation [18]. Monocytes express PD-L1, which when bound to T cells suppresses T cell proliferation and activation [18]. The mRNA-seq data showed that the monocyte PD-L1 expression was significantly increased after pEXO treatment (log_2_FC = 7.05) (Fig. 2A). The up-regulation of PD-L1 on pEXO-educated monocytes was confirmed by qPCR and flow cytometry (Fig. 5A, B). Consistently, PD-L1 was remarkably increased in monocytes of pregnant women compared to that of non-pregnant control of similar age (Fig. 5C).

Small RNA sequencing showed that miRNA was the largest group of non-coding RNA in the pEXOs (Additional file 1: Fig. S6A). A total of 75 miRNAs in pEXOs with > 5000 reads are shown in Additional file 4. Their miRNA target genes were predicted using sequence-based database tools, including Targetscan, Mirdb, Mirtarbase. We then conducted Gene Ontology (GO) analysis and Kyoto Encyclopedia of Genes and Genomes (KEGG) pathway analysis on the 570 predicted target mRNAs of the miRNAs in pEXOs. The target genes were mainly enriched in GO terms related to transcription and kinase/phosphatase activities (Additional file 1: Fig. S4B). The KEGG pathway analysis showed that the target genes of the miRNAs were primarily enriched in the miRNA in cancer, phosphatidylinositol 3-kinase (PI3K)-protein kinase B (AKT), mitogen-activated protein kinase (MAPK), Ras signaling pathway, etc. (Additional file 1: Fig. S4C).

Nine miRNAs were selected for validation based on high read count and literature review (Fig. 5D). miRNAs mimics were transfected into human monocytes respectively. The transfection efficiency was determined by FAM3™ dye-labelled pre-miR and the efficiency was ~ 45% (Additional file 1: Fig. S4C). Transfection of miR10a-5p, miR10b-5p, miR22-3p and miR29a-3p enhanced the PD-L1 expression in primary monocytes (Fig. 5E). Among the 17 common target genes of miR10-5p, miR22-3p and miR29a-3p predicated by TargetScan 7.2, PTEN is well known to be involved in down-regulating PD-L1 expression by inhibiting AKT phosphorylation (Fig. 5F) [19]. Of these miRNAs, the suppressive effect of miRNA-29a-3p on PTEN and PD-L1 expression in monocytes was confirmed by Western blot (Fig. 5G, H). Consistently, the monocytic PTEN levels of pregnant women were significantly lower when compared to that of non-pregnant controls (Fig. 5I, J). Together, our findings suggested that exosomal miRNA-29a-3p increased PD-L1 expression by down-regulating PTEN in monocytes.

## Discussion

**Fig. 6:**
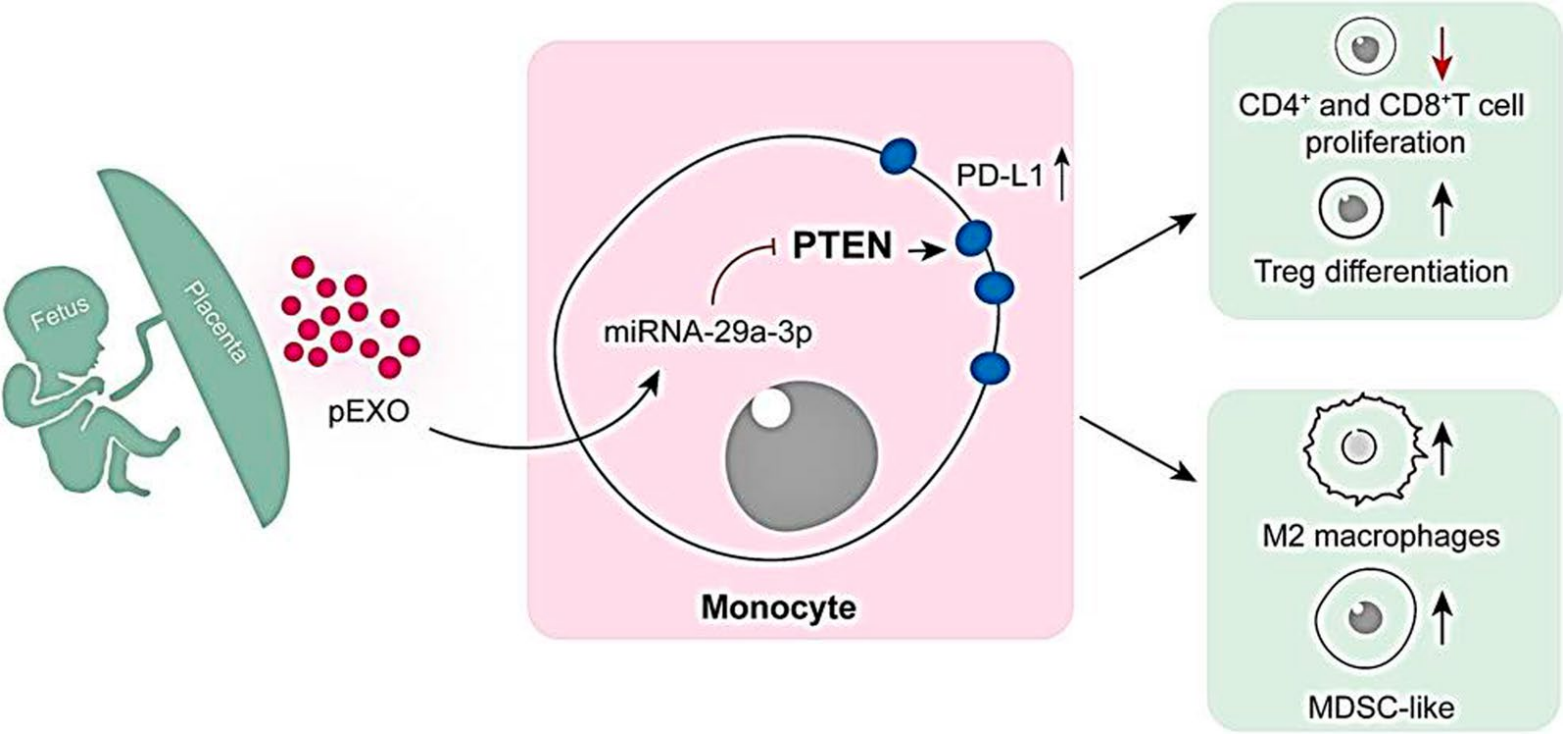
Schematic illustration showing the roles of pEXO on regulating maternal immune tolerance via reprogramming circulating monocytes. The maternal immune system needs to adapt to tolerate the semi-allogeneic conceptus. During early pregnancy, pEXO facilitates the establishment of systemic immune tolerance by transforming monocytes to an immunosuppression phenotype, including the MDSC subset. These pEXO-educated monocytes exhibited pleiotropic roles in promoting M2 macrophage polarization, suppressing T cell proliferation and inducing Treg cell differentiation

The maternal immune system is modified during pregnancy to tolerate the semi-allogeneic fetus [1]. The modifications of the immune system occur both at the maternal–fetal interface and the systemic circulation. How the maternal immune system tolerates the paternal antigens is still an enigma [1]. Here, we demonstrated that pEXOs induced maternal immunosuppression at systemic level by modulating monocytes and T cell phenotype/functions. More importantly, the pEXO effects were monocytes-dependent, indicating that the pEXO-educated monocytes are indispensable for maternal tolerance establishment in humans (Fig. 6). The observations are in line with reports showing that monocyte/macrophage depletion compromised reproductive performance in mice [20, 21].

The release of exosomes is an important mean of intercellular communication and exosomes can modulate lymphocyte and monocyte functions in different models [11, 12]. During pregnancy, pEXOs are mainly synthesized by syncytiotrophoblast via the lysosomal pathway and are released into the maternal circulation. The levels of circulating exosomes were 13.2-fold higher in pregnant than in non-pregnant women [22]. In this study, first trimester placenta explants were cultured under a hypoxia environment to imitate the in vivo condition [2] and pEXOs were isolated by differential ultracentrifugation. Compared to other pEXO isolation methods, the ultracentrifugation method is most widely used because of its consistency, reliability, easiness and high yield. Yet pEXOs isolated by different methods have unique biological effects [11]. The pEXOs in this study were characterized by three methods, namely expressions of specific markers, nanoparticle tracking analysis and electronic microscopy according to the International Society of Extracellular Vesicles guidelines [13]. However, the small size of the first trimester placental tissues obtained in the study forbid further characterization of the isolated pEXOs.

The regulatory role of exosomes in systemic immunotolerance has been implicated in cancers [23]. Of interest, monocytes are the primary cell type that internalizes cancer-derived exosomes in glioblastoma [24]. In our study, monocytes were the major cell type that interacted with pEXOs, and up to 95% of the monocytes uptook the PKH67-labelled exosomes. The pEXO-educated monocytes displayed immunosuppression phenotypes including expression of M-MDSC-associated markers, suppression of antigen-process and presentation associated genes and MHC class II molecules. Consistently, the frequency of immunosuppressive monocytes, characterized by CD14^+^HLA-DR^−/low^, was increased in early pregnancy [15] and reduced MDSC levels in blood and endometrium were found in miscarriage patients when compared to the normal pregnant control [25].

The innate immune responses changed during pregnancy [26]. The numbers of circulating innate immune cells (e.g. monocytes and granulocytes) are higher in pregnant than in non-pregnant women, resulting in an increased number of total leukocytes in the former [27]. The innate immune cells also show phenotypic and functional alternations during pregnancy. For instance, the circulating monocytes change their cytokine and oxygen radical production during pregnancy [26, 27]. Abnormal number or activities of monocytes/macrophages have been shown in several pregnancy complications [6, 7]. In preeclampsia, the monocytes are phenotypically and functionally activated as compared with those in normal pregnancy [27, 28].

In the current study, pEXO polarized macrophages to an M2-like phenotype with upregulation of M2 markers: CD163, CD206, CD209, IL-10 and IDO-1. GSEA and gene ontogeny analyses of the DEGs demonstrated that the pEXO-educated macrophages were similar to the IL-4/IL-13-treated M2a macrophages. Macrophages are broadly categorized into classically activated (M1) and alternatively activated macrophages (M2) [29]. Compared with M1 macrophages, M2 macrophages have immunosuppressive capacities and promote Th2 immune responses [30]. Although dMs belong to neither the M1 nor the M2 category [31], gene expression profiling of dMs shows an M2-dominant macrophages phenotype in the first trimester, characterized by expression of CD209 and anti-inflammatory genes [32]. The polarization of dMs to an anti-inflammatory state in early gestation is critical for pregnancy success [11] and activation of dMs towards an M1 phenotype is associated with recurrent miscarriages [33]. Given the importance of dMs in pregnancy, further investigation is warranted to elucidate the underlying mechanism regulating the differentiation of monocytes to dMs.

Treatment with pEXO also significantly increased the PD-L1 expression of monocytes. PD-L1 is a member of the B7-CD28 family and is a ligand of PD-1 [34]. From the 4th month of gestation, the PD-L1 expression is significantly enhanced in the placenta [35]. Recent studies demonstrate that the PD-L1/PD-1 pathway plays a role in establishing the unique phenotype of dMs, T cell homeostasis, peripheral tolerance and prevention of autoimmunity. Specifically, blocking the PD-L1/PD-1 pathway activates the PI3K/AKT/m-TOR and MEK/ERK signaling, changes the M2 phenotype of dMs to an M1 phenotype in vitro and in vivo [36], increases embryo resorption, abortions of allogeneic fetuses, reduces litter size, enhances expansion of Th1 cells, decreases number of Tregs and increase that of Th17 cells at the feto-maternal interface in mice [37]

Apart from the innate immune response, pregnancy-specific immune tolerance occurs in the T cells. The number of T cells is lower during pregnancy than before pregnancy [38]. Here we showed that the pEXO-educated monocytes remarkably reduced CD3/CD28 induced T cell proliferation. In addition, the levels of pro-inflammatory cytokines IFN-γ and TNF-α were reduced in the pEXO-educated monocytes, consistent with the impaired proliferation capacity. We also found that the inclusion of pEXO significantly reduced the proliferation of CD3^+^CD8^+^ cytotoxic T cells in comparison to the untreated control. The observations are in line with the observed changes in subsets and functional alterations of T-cell in pregnancy [38]. Compared to pre-pregnancy, the number of cytotoxic T cells was lower in the first trimester of pregnancy [39]. Upregulation of effector T cells, such as Th1 and Th17 cells, and deficiency of Tregs are associated with implantation failure, recurrent spontaneous abortion, and pre-eclampsia [3].

The pEXO-educated monocytes doubled the frequency of CD4^+^CD25^+^FoxP3^+^ Tregs in co-culture, suggesting that the circulating pEXO-educated monocytes contribute to Treg differentiation. Tregs, constituting about 5–15% of the peripheral CD4^+^ T cells [40], play a critical role in immune homeostasis and induction of maternal–fetal immunotolerance during pregnancy [41]. They strongly suppress the activation and proliferation of effector T cells and maintain immune tolerance by contact-dependent suppression or releasing anti-inflammatory cytokine IL-10 and TGF-b [42]. In human pregnancy, systemic and local expansion of the Treg pool occurs from the first trimester and peaks in the second trimester [43]. Abnormal function or a decreased number of Tregs is associated with pregnancy failure [44]. In mice, depletion of Tregs using anti-CD25 monoclonal antibodies induced implantation failure and abortion in allogenic pregnancies [45]. However, the origin of Treg cells in decidua and circulation during pregnancy is still unknown [46].

The action of the pEXO-educated monocytes on Tregs may be via increasing PD-L1 expression, which is necessary for the development of Tregs. One study showed that PD-L1 utilized Tregs to control maternal anti-fetal T cells in allogeneic pregnancy [47]. Furthermore, PD-L1 blockade reduces Tregs and increases the conversion of CD4^+^Foxp3^+^ into IL-17-producing T cells [48]. In contrast, PD-L1-coated beads (artificial antigen presenting cells) induce Tregs proliferation in vitro [18]. We speculate that PD-L1 was involved in pEXO-mediated T cell proliferation and elevated level of Treg cells. Interestingly, we observed that four miRNAs enriched in pEXO: miR10a-5p, miR10b-5p, miR22-3p, miR29a-3p could increase PD-L1 expression in monocytes, and that miR29a-3p down-regulated PTEN, the upstream regulatory of PD-L1 [49, 50]. Together, our data indicated that pEXO-mediated PD-L1 upregulation on monocytes by a miR29a-3p/PTEN/PD-L1 signaling pathway.

In summary, our study demonstrated that maternal monocytes facilitate the establishment of systemic immune tolerance by taking up pEXOs. The pEXO-educated monocytes promoted Treg differentiation by upregulation of PD-L1 on monocytes. Mechanistically, exosomal miRNA-29a-3p enhanced PD-L1 expression via the PTEN signaling pathway. These results uncover the importance of pEXOs in regulating the maternal systemic immune response during early pregnancy by reprogramming circulating monocytes. They form the basis for understanding the regulatory networks in the establishment of maternal immune tolerance to the fetal allograft.

In the long term, the study proposed that pEXOs might have the potential to serve as a biomarker for the diagnosis and treatment of immune-associated pregnancy complications. For example, preeclampsia patients have increased maternal blood levels of pEV-derived neprilysin [11, 12]. In addition, vaccination of immune cells treated with exosomes had shown promising effects for cancer treatment in mouse models [51].

## Methods

### Study subjects

Human placenta samples were collected from women undergoing surgical abortion in the first trimester (7–10 weeks) after written informed consent was obtained. The gestation age was determined by ultrasound assessment before the surgical abortion. After the operation, placental villi were collected, washed with sterile saline (0.9%) and transported to the laboratory on ice within 2 h. Blood samples from natural pregnant and nonpregnant women (age 25–40) were also collected with written consent for the project. The whole blood samples were collected into heparin tubes. Peripheral blood mononuclear cells were obtained by Ficoll-Hypaque gradient centrifugation (Sigma) according to the manufacturer’s protocol.

### Placental explant culture

Placenta tissues were washed with sterile PBS, the placenta villi were then removed from the chorion frondosum (villous chorion). A total of ~ 200 mg wet villi tissue was cultured in 40 µm cell strainer containing 7 ml DMEM/F12 medium supplemented with 5% exosome-free fetal bovine serum (EXO-FBS-250-1, SBI) and 1% penicillin–streptomycin/amphotericin B. The explants were cultured at 37 °C in a 2% oxygen environment, mimicking the in vivo environment of placenta development [2]. The culture medium was replaced after 3 h to remove the cell debris and apoptotic bodies, and the explants were cultured for a further 40 h to collect the pEXO.

### Isolation of placenta-derived exosomes

pEXO were collected by a standard serial centrifugation protocol (Fig. 1A) according to the International Society of Extracellular Vesicles guidelines [13]. In brief, conditioned media of placental villi were centrifuged at 300*g*, for 10 min to remove the dead cells. Cell debris was removed after centrifugation at 2000*g* for 20 min. Microvesicles were collected by centrifugation at 16,500*g* min at 4 °C for 30 min. Lastly, the supernatant after microvesicles collection was centrifuged at 120,000*g* for 120 min at 4 °C to obtain the pEXO. The isolated pEXO were washed with PBS twice, the protein concentration was measured by bicinchoninic acid (BCA) method, and stored at − 80 °C for future study.

### Characterization of purified exosomes

The morphology of the isolated pEXO was accessed using transmission electron microscopy (TEM). Briefly, pEXO were fixed in 4% PFA overnight and then dropped on formvar carbon-coated nickel grids. After the grids were stained with 4% uranyl acetate and 2% methylcellulose, air-dried and visualized using a Philip CM 100 transmission electron microscope (Electron Microscope Unit, The University of Hong Kong). The size and concentration of the pEXOs were analyzed by NTA using ZetaView (Particle Metrix, Meerbusch, Germany).

For characterization of the exosome markers, pEXO proteins (20 μg per well) were resolved in 10% SDS-PAGE for Western blot analysis using primary antibodies against CD63, CD81, HSP70 in appropriate dilutions (Additional file 1: Table S1).

### Non-coding RNA sequencing

pEXO from five patients were pooled together. The miRNAs were extracted by mirVanaTM RNA Isolation kit (Thermo Fisher Scientific) according to the manufacturer’s protocol. The quality of small RNAs was determined by Nanodrop2000 (Thermo Fisher Scientific). The samples were submitted to Centre for PanorOmic Sciences, The University of Hong Kong for library preparation and sequencing. Briefly, 100 ng of small RNA was used as the template. Libraries were prepared by the NEBNext^®^ Multiplex Small RNA Library Prep Set for Illumina (New England Biolabs, MA, USA).

Sequencing reads were first filtered for adapter sequences and low-quality sequences followed by retaining reads with a read length ≥ 15 bp. Filtered reads were mapped to the mature miRNA sequences (miRBase v21) and unmapped reads were further mapped to hairpin miRNA sequences (miRBase v21). Subsequently, leftover reads were mapped to piRNA sequences (RNAcentral) and rRNA sequences. Bowtie2 was used for the alignment using default parameters for strand-specific RNASeq. The biological functions of miRNAs were by mirPath v3.0, TargetScan 7.2 and Starbase 2.0.

### Exosome interaction

pEXO were labelled with the CellTrace™ CFSE (Thermo Fisher) according to the manufacturer’s protocol. Interaction of the CFSE-labelled pEXO by peripheral blood mononuclear cells for 24 h was analyzed by flow cytometry.

### Immune cell isolation

Human monocytes were purified from the buffy coat of healthy female donors by positive immunomagnetic selection, using CD14^+^ microbeads, according to the manufacturer’s instructions (Miltenyi Biotec). CD14 binding does not activate monocytes since it lacks a cytoplasmatic domain. Autologous human T cells were purified from the sample donor using immune-depletion on a Ficoll-Hypaque gradient (RosetteSep, Stemcell Technology) according to the manufacturer’s protocol. The enriched monocytes and T cells were resuspended in RPMI-1640 medium supplemented with 10% FBS.

### pEXO treatment

Monocyte (1 × 10^6^ cells) were treated with pEXO (20 μg) for 24 h in our study and used for qPCR and flow cytometry analysis.

### pEXO-polarized macrophages

M-CSF is highly expressed in decidua and promotes dM and M2 polarization [16]. Macrophages (2 × 10^6^ cells were differentiated from monocytes by treatment with 50 ng/ml M-CSF (BioLegend, San Diego, CA, USA) for 7 days. The cells were further incubated with pEXO (20 µg/ml) on the 7th day for 24 h.

### pEXO-educated monocyte/T cell co-culture

T cells (2 × 10^5^ cells) were cocultured with monocytes in a ratio of 1:1 and were stimulated with CD3/CD28 microbeads, IL-2 (30 UI/ml) for 3 days. pEXO or pEXO-educated monocytes were added to the co-culture system. To study the potential suppressive effector of T cell proliferation, monocytes (2 × 10^5^ cells/100 μl) or monocyte spent medium (100 μl) were added to the autologous T cells and cultured for 3 days. CFSE dye was used for tracking cell division according to the manufacturer’s protocol (Thermo Fisher Scientific). In brief, 1 × 10^6^ T cells were labelled with 5 mM CFSE in PBS for 20 min and the reaction was stopped by the RPMI-1640 medium supplemented with 10% FBS for flow cytometric analysis. Unstimulated CFSE-labelled T cells were used as non-dividing control.

### Reverse transfection of human monocytes with miRNA mimics

miRNA mimics transfection was performed using Lipofectamine RNAiMAX reagent. Briefly, monocytes (1 × 10^6^ cells/ml) were gently mixed with the miRNA mimics-Lipofectamine RNAiMAX mixture and incubated for 24 h. Fluorescent pre-miNRAs (FAM3™ dye-labelled, ThermoFisher, USA) were used as control. The transfection efficiencies were tested by flow cytometry.

### Flow cytometry analysis

Cells were washed with ice-cold PBS after the treatments. For antibody staining, Fc receptors of monocytes were blocked with the Fc block (Biolegend) to avoid non-specific binding. Membrane proteins were stained for 30 min at 4 °C. Intracellular proteins were stained for 60 min at 4 °C after treatment with commercial fixation/permeabilization reagents (eBioscience) (Additional file 1: Table S1). For the apoptosis assay, T cells were stained with YO-PRO-1 (Thermo Fisher Scientific) according to the manufacturer’s instructions. All flow cytometric measurements were performed by Cytoflex (Beckman coulter) and data were analyzed by flowJo v10 (Tree Star, Inc., Ashland, OR, USA).

### Enzyme-linked immunosorbent assay (ELISA)

The conditioned media of co-culture of pEXO-educated monocytes with T cells were collected. The level of IFN-γ, TNF-α, IL-10 and TGF-β in the media were quantified by ELISA kits following the manufacturer’s instruction (eBioscience).

### Quantitative PCR (qPCR)

Total RNA was isolated from CD14^+^ monocytes using the illustra™ RNAspin mini kit (GE), and reverse transcribed into first-strand complementary DNA (cDNA) with random primer using the PrimeScript RT reagent kit (Takara). The samples were then analyzed in an Applied Biosystems QuantStudio5 Real-Time PCR system (Thermo Fisher Scientific). 18S ribosomal RNA was used as the internal control. For the primer information, please refer to Additional file 1: Table S2.

### mRNA-seq and bioinformatic analysis

Human monocytes were collected for mRNA isolation after pEXO treatment for 24 h. The quality of the isolated mRNA was determined by Nanodrop 2000. All samples were submitted to BGI Genomics (BGI Group, Shenzhen, China) for mRNA sequencing. The quality of raw sequencing data was first assessed using the fastQC (version 0.11.8), before cleaning the data by the fastp (version 0.20.0). Low-quality reads whose phred quality ≤ Q15 were removed. The clean reads were aligned to the human reference genome (hg38) using the Hisat2 (version 2.1.0). Gene counts were calculated by the featureCounts (version 1.6.4) [52]. To analyze the differential gene expression, the gene-level read count matrix was then imported into the R (version 4.0.3). In this process, the DEseq2 (verst1sion 1.30.0) [53], edgeR (version 3.32.0) [54] and limma (version 3.46.0) [55] packages were used. DEGs with raw p-value < 0.001 and fold change contrast ≥ 16 from each package were preserved. The intersect of the filtered DEGs from each package were selected as interested DEGs for each group comparison, with functional analysis (GO and KEGG analysis) by the clusterProfiler (version 3.18.1) [56]. Besides, the DEGs were used to plot heatmaps by the pheatmap (version 1.0.12) with scaling value of the normalized read count matrix. The analyzed data were deposited in the NCBI GEO database under the accession code GSE184195.

### Statistical analysis

Data were expressed as mean ± standard deviation (SD), and analyzed by the Graph Pad Prism 7 (Graph Pad Software Inc., CA, USA). All the data were analyzed by the Kolmogrov–Smirnov normality test followed by either the non-parametric Mann Whitney U test or the parametric Student’s t-test. p < 0.05 was considered as a significant difference.

## Supplementary Information

**Additional file 1.** Supplementary research data of human placental exosomes induce maternal systemic immune tolerence by reprogramming circulating monocytes.

**Additional file 2. **DEGs between control and pEXO-educated monocytes.

**Additional file 3. **DEGs between control and pEXO-polarized macrophages.

**Additional file 4.** MiRNA content of the pEXOs.

## Data Availability

All data needed to evaluate the conclusions in the paper are present in the paper or the Additional files. The mRNA-seq data were deposited in the NCBI GEO database (https://www.ncbi.nlm.nih.gov/geo/), under the accession code GSE184195.

## Abbreviations

BCA: Bicinchoninic acid
CDSE: Carboxy-fluorescein succinimidyl ester
DM: Decidual macrophage
DC cell: Dendritic cell
DEGs: Differentially expressed genes
FGR: Fetal growth restriction
GO: Gene Ontology
GSEA: Gene set enrichment analysis
HLA: Human leukocyte antigen
IL: Interleukin
M-CSF: Macrophage colony-stimulating factor
MHC: Major histocompatibility complex
M-MDSC: Monocytic myeloid-derived suppressor cells
NTA: Nanoparticle tracking analysis
NK cell: Natural killer cells
pEXO: Placenta-derived exosomes
PD-L1: Programmed cell death ligand-1
PTEN: Phosphatase and tensin homolog
Treg: Regulatory T cells
Th: T helper cell type
