# Supplementary material for: Human placental exosomes induce maternal systemic immune tolerance by reprogramming circulating monocytes
Source: J Nanobiotechnology. 2022 Feb 18;20:86. doi: 10.1186/s12951-022-01283-2 (PMC8857816; doi:10.1186/s12951-022-01283-2)

Additional file 1: Table S1. Antibodies used for flow cytometry

Antibodies	Identifier	Resource	Volume / reaction
Pacific Blue™ anti-human CD4 Antibody	317429	Biolegend	1 µl
PE anti-human CD223 (LAG-3) Antibody	369305	Biolegend	5µl
APC anti-human CD152 (CTLA-4) Antibody	349908	Biolegend	5µl
FITC anti-human CD73 (Ecto-5'-nucleotidase) Antibody	344016	Biolegend	5µl
APC/Cyanine7 anti-human CD11c Antibody	337217	Biolegend	5µl
PE anti-human CD56 (NCAM) Antibody	318305	Biolegend	5µl
Alexa Fluor® 700 anti-human CD19 Antibody	302225	Biolegend	5µl
Brilliant Violet 605™ anti-human CD3 Antibody	317321	Biolegend	5µl
APC anti-human HLA-DR, DP, DQ Antibody	361713	Biolegend	5µl
APC/Cyanine7 anti-human CD273 (B7-DC, PD-L2) Antibody	345515	Biolegend	5µl
Pacific Blue™ anti-human CD14 Antibody	367121	Biolegend	2µl
Human TruStain FcX™ (Fc Receptor Blocking Solution)	422302	Biolegend	5µl
PE anti-human CD8	344706	Biolegend	5µl
FITC anti-human FOXP3	320106	Biolegend	5µl
PE anti-human CD25	302606	Biolegend	5µl

Additional file 1: Table S2. Primers used for RT-qPCR

Primers	Resource	Identifier
CD163	Thermo Scientific	Hs00174705_m1
CD206	Thermo Scientific	Hs00267207_m1
CD209	Thermo Scientific	Hs01588349_m1
CD274	Thermo Scientific	Hs00204257_m1
CCL2	Thermo Scientific	Hs00234140_m1
CCL8	Thermo Scientific	Hs04187715_m1
IL10	Thermo Scientific	Hs00961622_m1
IDO1	Thermo Scientific	Hs00961622_m1
HLA-DRA	Thermo Scientific	Hs00984148_m1
18s	Thermo Scientific	4319413E

Additional File 1 Supplementary Fig. S1. Flow cytometry gating strategy of different immune cell populations. Immunophenotyping using the classical immune cell surface markers. CD3 for T cells, CD14 for monocytes, CD19 for B cells, CD56 for NK cells, and CD11c for dendritic cells.

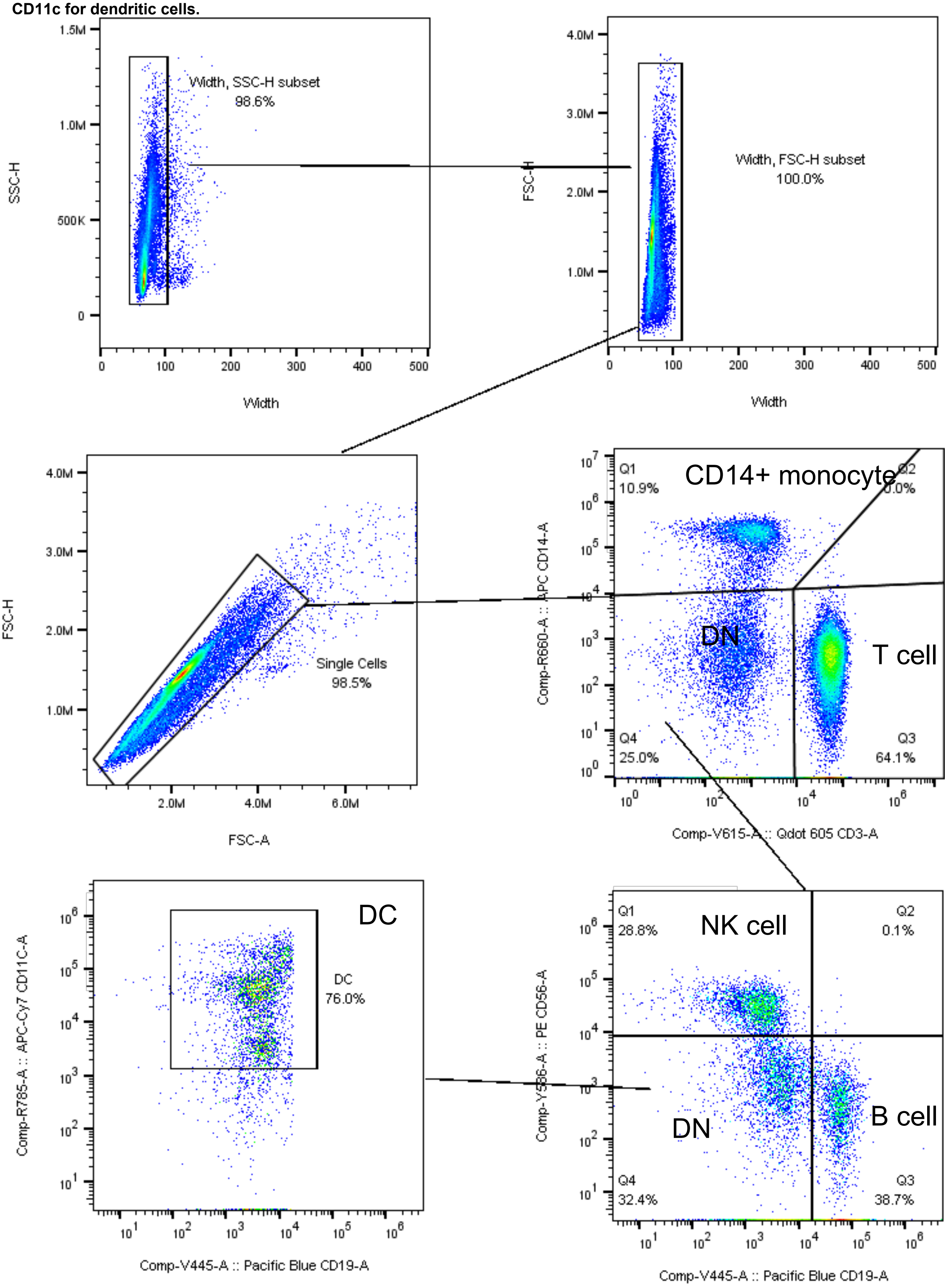

Additional File 1 Supplementary Fig. S2. Functional categories of pEXO-educated monocytes. (A-D) Heatmaps of genes that have significant changes in pEXO-educated monocytes compared with untreated controls were clustered according to the corresponding functional categories.

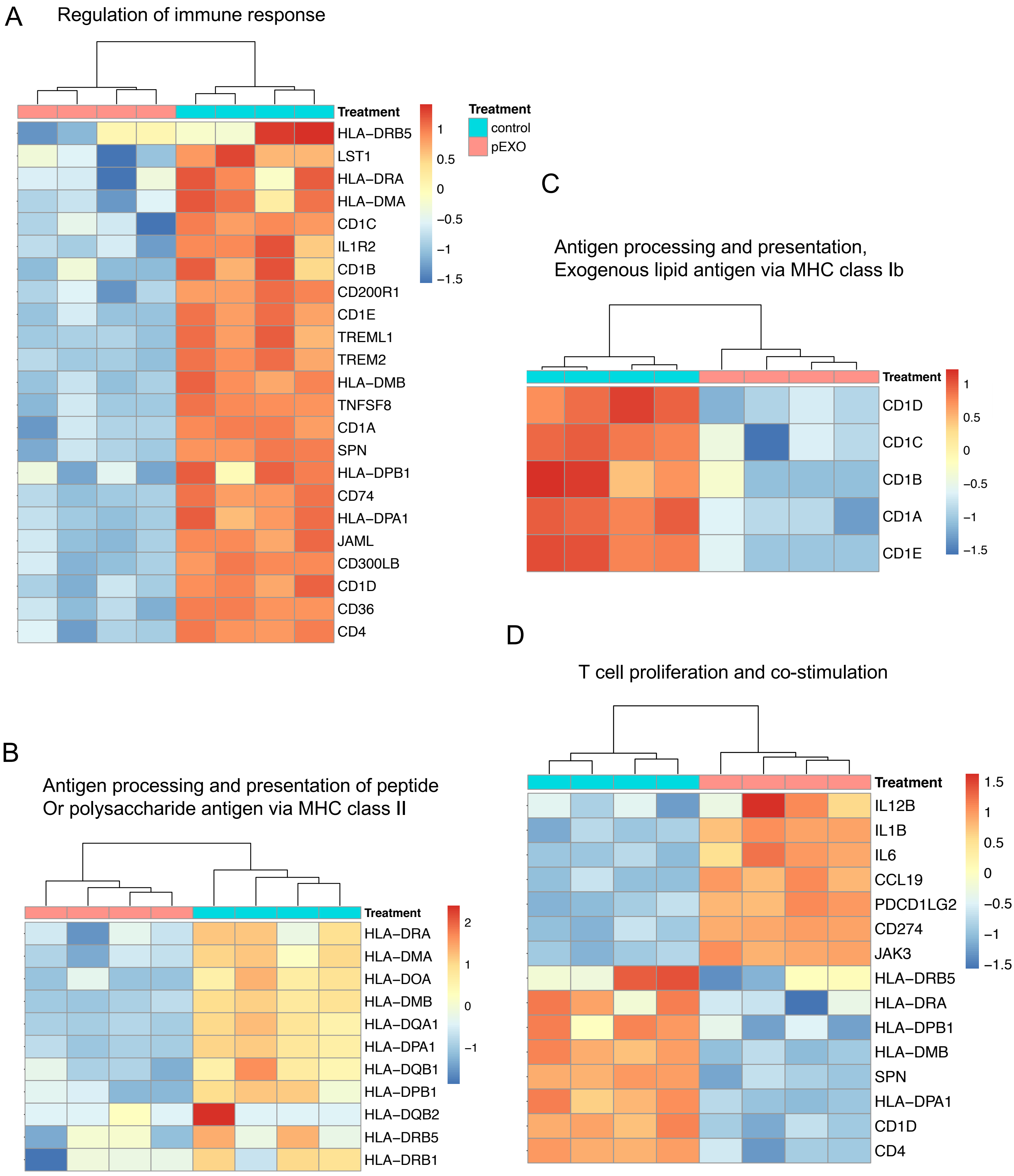

Additional File 1 Supplementary Fig.S3. Autologous monocytes do not influence T cell proliferation. CD14+ monocyte with or without pEXO (20 μg/ml) treatment were allowed to co-culture with the CD4+/CD8+ T cells isolated by MACS from the same donor at a ratio(1:1) for 4 days. CFSE dye was then used for tracking cell division according to the manufacturer's protocol. Representative histogram of CFSE-labelled human T cells were shown. The percentage of CFSE-labelled human T cells were presented as mean±SD (N=4). **P<0.05 when compared to the control T cells without monocyte coculture.

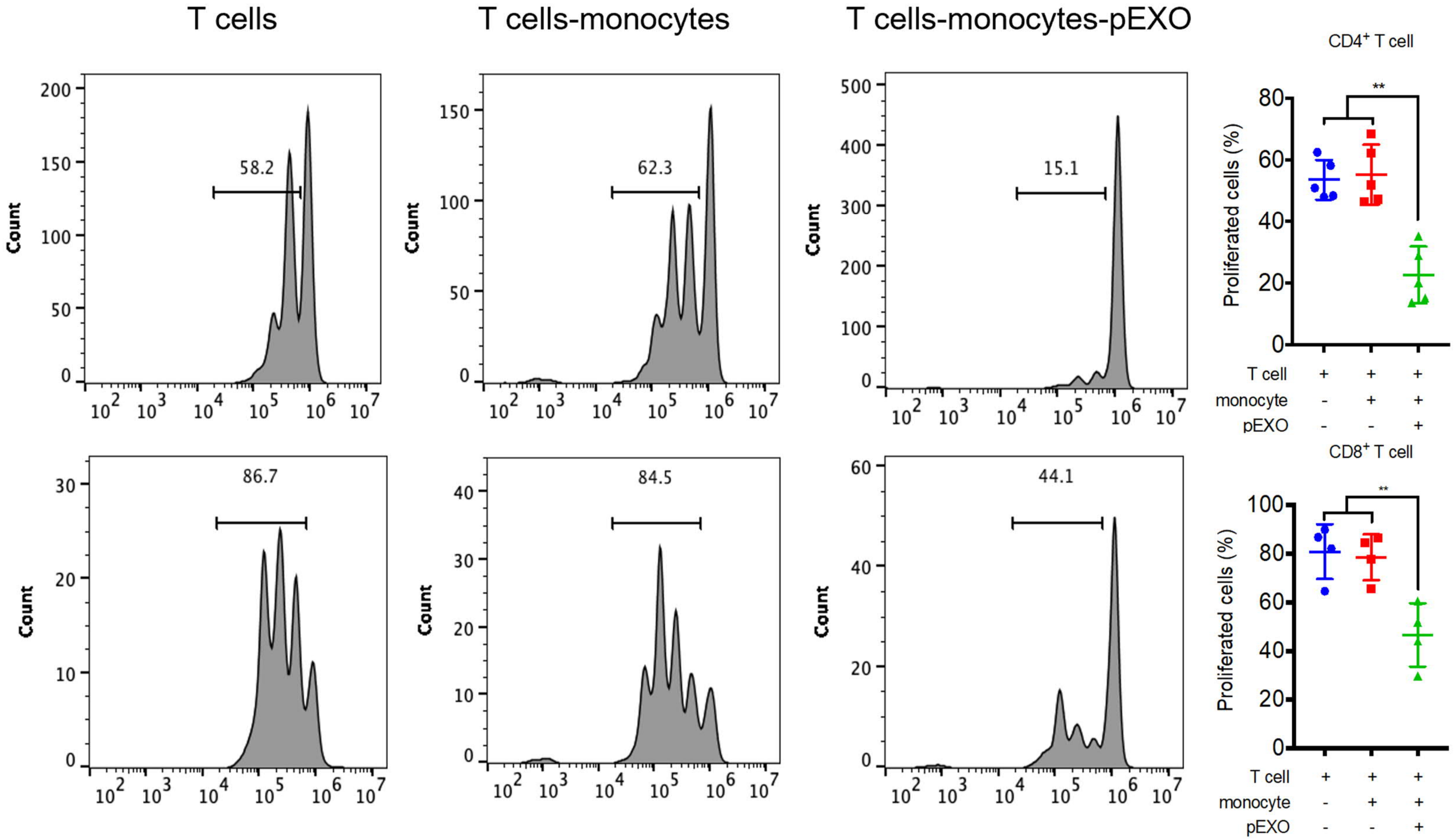

Additional File 1 Supplementary Fig. S4. pEXO did not affect the viabilities of human monocytes and T-cells. (A) Effect of pEXO on viability of monocytes by XTT assay. Monocytes were incubated with 0-50 μg/ml of pEXOs for 24 hours. XTT signals were analyzed by the ELISA spectrophotometer at. 450 nm. Representative flow cytometry plots were also shown. (B) Viable, necrotic and apoptotic T cells after 20 μg pEXO treatment for 24 hours were quantified by bivariate Yo-Pro-1/PI flow cytometry. Representative flow cytometry plots and the data of viable, necrotic and apoptotic T-cells were shown. All the values were presented as mean±SD (N=3). No significant difference was detected when compared to the control group.

A

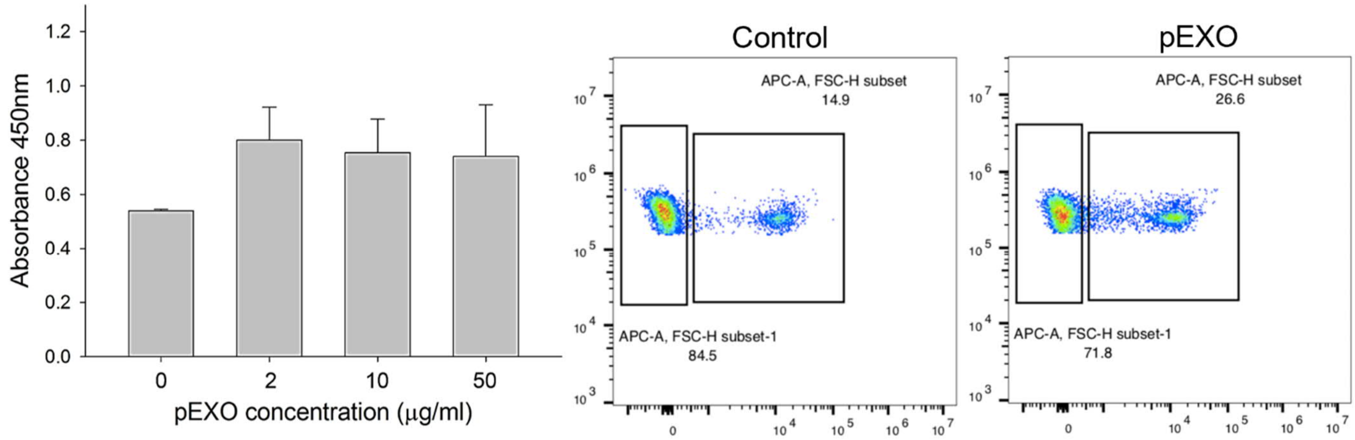

B

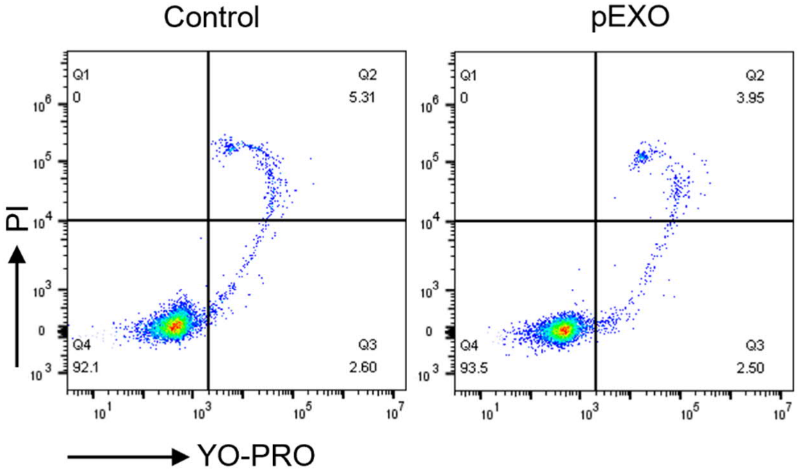

	Viable	Apoptosis	Dead
Control	92.7 ± 0.94	2.265 ± 0.34	5.04 ± 0.65
pEXO	93 ± 0.75	2.72 ± 0.3	4.24 ± 0.58

Additional File 1 Supplementary Fig. S5. Non-coding RNA-seq of pEXO. (A) Reads distribution of small RNAs in pEXOs. (B) TOP 20 enriched GO terms of miRNA target genes. (C) The target genes of miRNAs in PEXOs were analyzed for KEGG pathway enrichment. (D) The transfection efficacies of miRNA mimics were examined by flow cytometry. Values were presented as mean±SD; ****p<0.001 when compared to the control group.

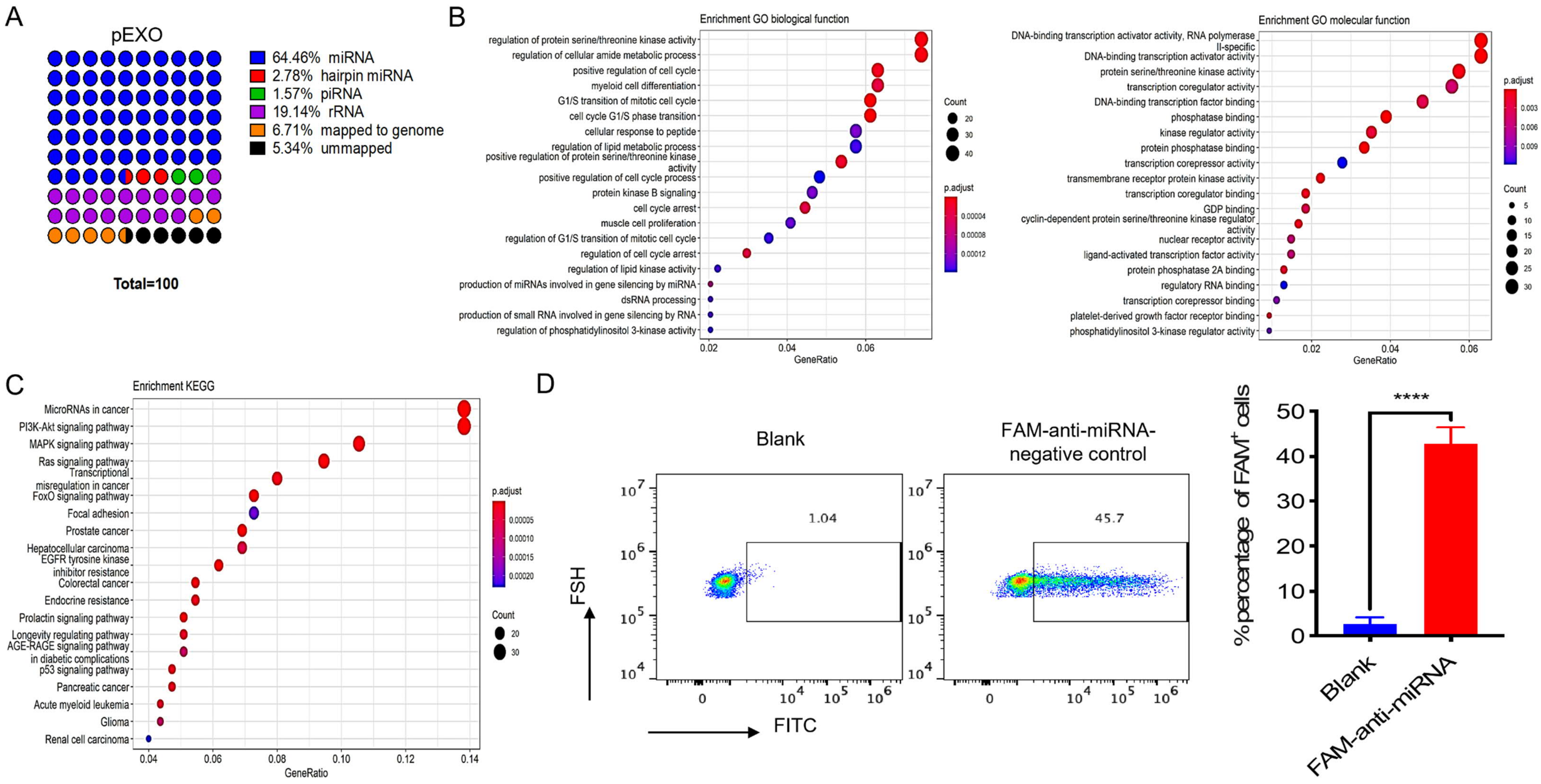

Supplement: Supplementary file 1 — Additional file 1. Supplementary research data of human placental exosomes induce maternal systemic immune tolerence by reprogramming circulating monocytes. [file 12951_2022_1283_MOESM1_ESM.pdf]
